# Peripheral perfusion index predicting prolonged ICU stay earlier and better than lactate in surgical patients: an observational study

**DOI:** 10.1186/s12871-020-01072-0

**Published:** 2020-06-18

**Authors:** Xinge Shi, Ming Xu, Xu Yu, Yibin Lu

**Affiliations:** Department of Critical Care Medicine, Xinyang Central Hospital, No.1, Siyi Road, Xinyang, 464000 Henan Province China

**Keywords:** Peripheral perfusion index, Surgical patients, Critical patients, Hypoperfusion, Lactate, Length of ICU stay

## Abstract

**Background:**

Peripheral perfusion index (PPI) is an indicator reflecting perfusion. Patients undergoing long time surgeries are more prone to hypoperfusion and increased lactate. Few studies focusing on investigating the association between PPI and surgical patients’ prognoses. We performed this study to find it out.

**Methods:**

From January 2019 to September 2019, we retrospected all surgical patients who were transferred to ICU, Xinyang Central hospital, Henan province, China. Inclusive criteria: age ≥ 18 years old; surgical length ≥ 120 min. Exclusive criteria: died in ICU; discharging against medical advice; existing diseases affecting blood flow of upper limbs, for example, vascular thrombus in arms; severe liver dysfunction.

We defined “prolonged ICU stay” as patients with their length of ICU stay longer than 48 h. According to the definition, patients were divided into two groups: “prolonged group” (PG) and “non-prolong group” (nPG). Baseline characteristics, surgical and therapeutic information, ICU LOS, SOFA and APACHE II were collected. Besides we gathered data of following parameters at 3 time points (T0: ICU admission; T1: 6 h after admission; T2: 12 h after admission): mean artery pressure (MAP), lactate, heart rate (HR), PPI and body temperature. Data were compared between the 2 groups. Multivariable binary logistic regression and ROC (receiver operating characteristic) curves were performed to find the association between perfusion indictors and ICU LOS.

**Results:**

Eventually, 168 patients were included, 65 in PG and 103 in nPG. Compared to nPG, patients in PG had higher blood lactate and lower PPI. PPI showed significant difference between two groups earlier than lactate (T_0_ vs T_1_). The value of PPI at two time points was lower in PG than nPG(T0: 1.09 ± 0.33 vs 1.41 ± 0.45, *p =* 0.001; T1: 1.08 ± 0.37 vs 1.49 ± 0.41, *p <* 0.001).

Increased lactate_T1_(OR 3.216; 95% CI 1.253–8.254, *P =* 0.015) and decreased PPI_T1_ (OR 0.070; 95% CI 0.016–0.307, *P* < 0.001) were independently associated with prolonged ICU stay. The area under ROC of the PPI_T1_ for predicting ICU stay> 48 h was 0.772, and the cutoff value for PPI_T1_ was 1.35, with 83.3% sensitivity and 73.8% specificity.

**Conclusions:**

PPI and blood lactate at T_1_(6 h after ICU admission) are associated with ICU LOS in surgical patient. Compared to lactate, PPI indicates hypoperfusion earlier and more accurate in predicting prolonged ICU stay.

## Introduction

Hemodynamic changes in postoperative patients are complex and various. Many patients would manifest increased level of blood lactate after long-term surgeries [1–3]. Ensuring adequate perfusion is essential for recovery and better prognosis.

Many indicators have been proved reliable in reflecting perfusion. Among them, blood lactate is one of the widest used indexes guiding fluid management and resuscitation [4, 5]. But it still has some drawbacks. The most evident one is lactate is delayed in reflecting real-time perfusion. As we all know, from the beginning of hypoperfusion to elevated lactate, there is a time interval. If we use lactate as an indicator of hypoperfusion, we will take measures later than the real start of inadequate perfusion, which is why we turn to a more timely parameter: peripheral perfusion index (PPI).

Some researchers have proved PPI is a reflector of hypoperfusion [6, 7] and is associated with patients’ mortality [8]. But most of these studies are about septic shock. Apart from that, no study has been about comparing lactate with PPI, trying to figure out which one is superior in predicting patients’ outcomes.

If we can prove PPI is comparable or superior to lactate in reflecting postoperative patients’ prognoses, we will take beneficial interventions earlier because PPI is more instant than lactate in mirroring perfusion state. That is why we performed this study to investigate the role of PPI in predicting postoperative patients’ prognoses and to compare PPI with lactate, finding out their strengths and limitations.

## Patients and methods

### Patients

The Institutional Research and Ethics Committee of the Xinyang Central Hospital approved this study. Written informed consent was waived since it was a retrospective study.

This was a retrospective study of all surgical patients who were admitted to ICU in Xinyang Central Hospital (Henan province, China) from January 1st, 2019 to September 30th, 2019. The inclusive criteria are following:1) age ≥ 18 years old; 2) surgical length ≥ 120 min. Exclusive criteria: 1) died in ICU; 2) discharging against medical advice; 3) existing diseases affecting peripheral blood flow, for example, vascular thrombus in arms; 4) severe liver dysfunction.

Prolonged ICU stay was defined as ICU LOS longer than 48 h (from the time of arrival in ICU). Patients were divided into two groups according to the definition: “prolong group” (PG) and “non-prolong group” (nPG).

### Data collection

Before we commenced data collection, we had estimated the minimum sample size we needed to obtain (supplement.1). The electronic medical records and anaesthesia charts of all recruited patients were reviewed to collect the following information: gender, age, comorbidities(CAD: coronary artery disease; COPD: chronic obstructive pulmonary disease; CKD: chronic kidney disease; HTN: hypertension; PMI: pacemaker implantation), surgical types and length, volume of intraoperative fluid infusion, fluid balance of 0-6 h and 0-12 h in ICU, the application of vasoactive agent in the first 12 h in ICU, SOFA(Sequential Organ Failure Assessment), APACHE II(Acute Physiology and Chronic Health Evaluation) on 1st day in ICU and the length of ICU stay.

We also collected each patient’s perfusion associated parameters at three different time points: ICU admission(T0), 6 h after ICU admission(T1), 12 h after ICU admission(T2). The perfusion related variables include mean artery pressure (MAP), heart rate (HR), peripheral perfusion index (PPI) and body temperature (axillary). MAP, HR and PPI would be obtained by monitors continuously (Philips IntelliVue MP50; Koninklijke Philips, The Netherlands) and exported to our medical records database automatically. PPI was captured by the pulse oximeter and blood pressure was measured using the oscillometric non-invasive technique or via radial arterial cannulation using pulse contour analysis.

As for PPI, we used its absolute value in every 15 min to calculate the averaged value for each hour. All PPIs were averaged values (in the first, sixth and twelfth hour in ICU).

### Statistical analysis

This is a retrospective study only involving in-hospital information. We assumed missing data were limited to a small number of observations and adopted likewise deletion to handle missing data.

Descriptive analyses were performed for the nPG and PG groups. Continuous variables are expressed as means ± standard deviations, and categorical variables are expressed as absolute values and percentages. For the continuous variables, the data were analyzed using Student’s t-test, the Mann-Whitney U test or the Kruskal-Wallis test depending on the data distribution and the number of variables. The categorical variables were analyzed using chi-square or Fisher’s exact tests.

Before we performed the multivariate logistic regression analysis, univariate analysis was performed for each potential risk factor of prolonged ICU stay. We defined *P <* 0.05 in univariate analyzation as the threshold of a variable qualified to be included in multivariate logistic regression model. As for indicators associated with prolonged ICU stay, the receiver operating characteristic (ROC) analyses were performed to test each indicator’s efficacy in predicting prolong ICU stay. All comparisons were two-tailed, and *P <* 0.05 was required to exclude the null hypothesis. The statistical analysis was performed using IBM SPSS Statistics, Version 20.0 (Armonk, NY: IBM Corp).

## Results

**Fig. 1:**
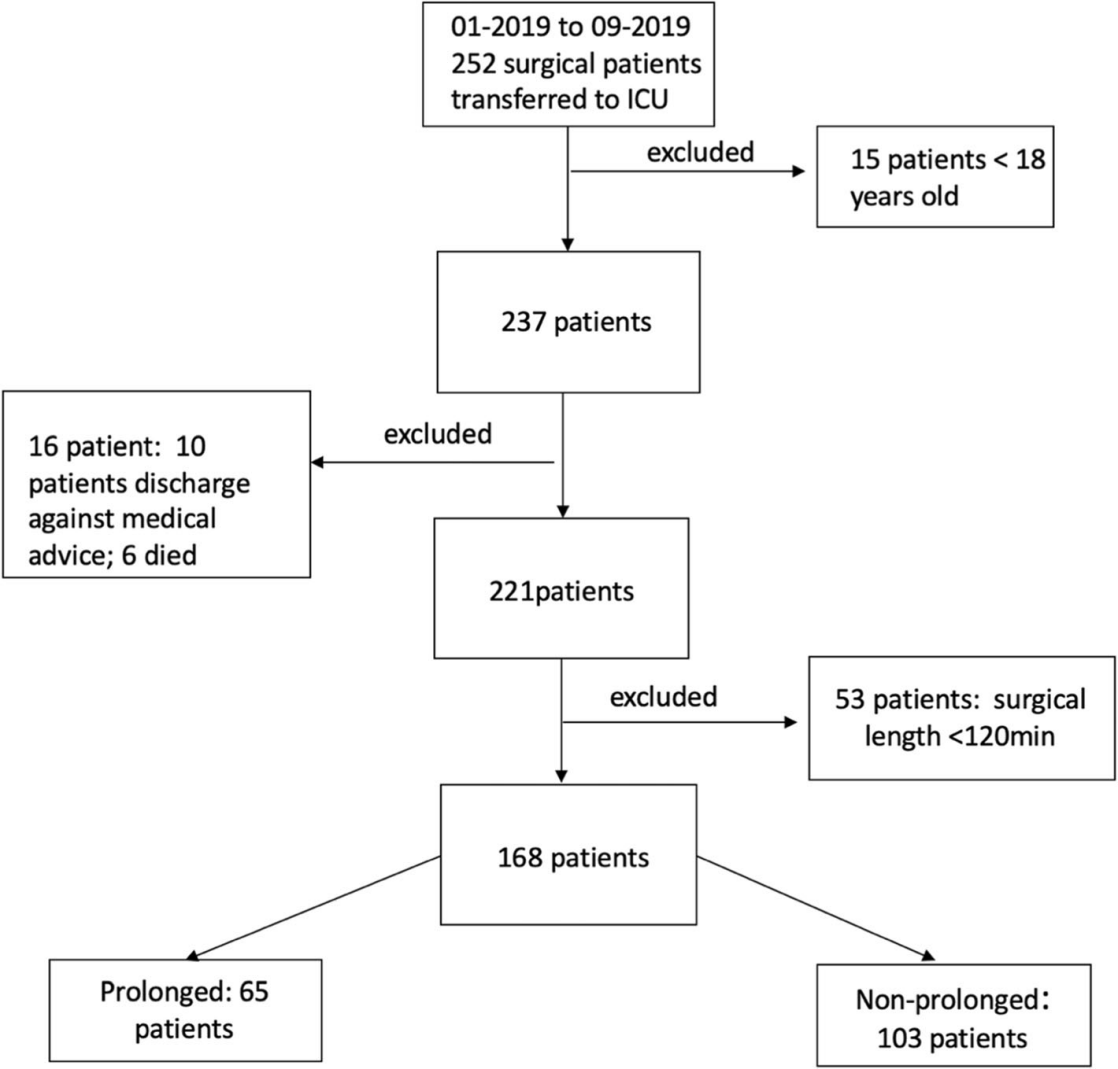
Diagram showing the enrollment of patients

From January to September 2019, a total of 252 surgical patients were transferred to ICU, Xinyang Central Hospital, Henan Province, China. 84 patients were excluded for several reasons and 169 patients were recruited finally. They were divided into 2 groups according to their ICU LOS: 65 patients in “prolonged group”, 103 patients in “non-prolonged group”. Main reasons for each patient in PG were showed in supplement 2. The ICU LOS was 66.63 ± 13.48 h in PG and 35.21 ± 8.19 h in nPG, *P <* 0.001. The flow chart of patients’ enrollment was showed in Fig. 1.

### Baseline characteristics

We analyzed the basic characteristics (age, gender, APACHEII and SOFA on 1st day) between the two group, and no significant differences were observed. We calculated the number of patients who have different comorbidities (CAD: coronary artery disease; COPD: chronic obstructive pulmonary disease; CKD: chronic kidney disease; HTN: hypertension; PMI: pacemaker implantation) and no differences were found between the 2 groups. More details were displayed in the Table 1.

**Table 1:** The baseline characteristics of enrolled patients

		Prolonged ICU stay *N* = 65	Non-prolonged ICU stay *N* = 103	*P*
Age /year		54.96 ± 11.22	57.37 ± 10.51	0.245
Gender male/%		50/76.92%	68/66.02%	0.132
LOS ICU /h		66.63 ± 13.48	35.21 ± 8.19	< 0.001
APACHE II		12.32 ± 1.48	12.09 ± 1.66	0.276
SOFA		5.09 ± 1.43	4.97 ± 1.36	0.661
Comorbidity	CAD	16	22	0.763
	COPD	10	9	0.282
	CKD	7	6	0.383
	HTN	20	29	0.850
	Diabetes	10	16	1.000
	PMI	7	8	0.699
	Others	3	2	0.329

*LOS ICU* length of ICU stay, *APACHE II* Acute Physiology and Chronic Health Evaluation, *SOFA* Sequential Organ Failure Assessment, *CAD* coronary artery disease, *COPD* chronic obstructive pulmonary disease, *CKD* chronic kidney disease, *HTN* hypertension, Others: atrial fibrillation, stroke, asthma

### Surgical and therapeutic information

The surgical length of PG and nPG was 208.78 ± 63.58 min and 197.72 ± 54.33 min, respectively, with *P* value being 0.100. For prolonged and non-prolonged patients, amount of intraoperative fluid infusion was 1687.86 ± 465.75 ml and 1575.00 ± 419.97 ml(*P =* 0.295) while amount of fluid input in the first 24 h in ICU was 2357.90 ± 409.88 ml and 2115.01 ± 238.77 ml(*P =* 0.006), respectively. Considering types of surgery could influence postoperative perfusion, we collected data of surgical types (abdomen, chest, lung, esophagus, gynecology, great artery, others). None of them were significantly different between two groups. Vasoactive agent is another factor influencing peripheral prefusion. The number of patients with application of vasoactive drugs in the 1st 12 h in ICU were 7 out of 65 in PG and 15 out of 103 in nPG (*P =* 0.478). More details were displayed in the Table 2.

**Table 2:** Surgical and therapeutic information

		Prolonged ICU stay *N* = 65	Non-prolonged ICU stay *N* = 103	*P*
Surgical length /min		218.63 ± 60.35	195.83 ± 54.84	0.100
Intraoperative fluid infusion /ml		1687.86 ± 465.75	1575.00 ± 419.97	0.295
Fluid balance /mL	0-6 h	296.00 ± 151.88	280.24 ± 110.44	0.611
	0-12 h	395.67 ± 174.29	454.29 ± 184.70	0.179
Vasoactive agent n(%)		7(10.77%)	15(14.56%)	0.478
Operative types	Abdominal	21	38	0.660
	Chest	8	16	0.722
	Lung	10	10	0.389
	Esophagus	7	14	0.765
	GO	10	12	0.643
	GA	9	13	1.000

*GO* gynecological oncology, *GA* great artery

**Table 3:** Comparison of hemodynamic and perfusion parameters between the two groups

		Prolonged ICU stay *N* = 65	n1	Non-prolonged ICU stay *N* = 103	n2	P
HR bpm	T_0_	88.90 ± 9.52	65	89.45 ± 10.39	103	0.819
	T_1_	82.87 ± 5.88	65	83.50 ± 5.45	103	0.640
	T_2_	80.40 ± 7.44	65	80.81 ± 6.51	103	0.805
MAP /mmHg	T_0_	74.52 ± 6.83	65	76.28 ± 8.33	103	0.221
	T_1_	75.33 ± 4.86	65	75.49 ± 5.21	103	0.864
	T_2_	74.74 ± 5.49	65	74.58 ± 5.62	103	0.883
Lactate mmol/L	T_0_	3.08 ± 0.72	65	3.09 ± 0.73	103	0.978
	T_1_	3.13 ± 0.67	58	2.72 ± 0.59	89	0.009
	T_2_	3.15 ± 0.44	61	2.02 ± 0.42	92	0.001
PPI	T_0_	1.09 ± 0.33	65	1.41 ± 0.45	103	0.001
	T_1_	1.08 ± 0.37	65	1.49 ± 0.41	103	< 0.001
	T_2_	1.17 ± 0.27	65	1.23 ± 0.33	103	0.392
T / °C	T_0_	36.45 ± 0.33	65	36.46 ± 0.35	103	0.875
	T_1_	36.84 ± 0.20	65	36.81 ± 0.21	103	0.422
	T_2_	37.02 ± 0.42	65	36.98 ± 0.41	103	0.626

*HR* heart rate, *MAP* mean artery pressure, *PPI* peripheral perfusion index, *T* temperature

### Hemodynamic and perfusion indicators

At three time points (T_0_, T_1_, T_2_), we did not see any differences on HR, MAP and body temperate between the 2 groups. When patients arrived in ICU, there were no differences on the immediate (T_0_) lactate between PG(3.08 ± 0.72 mmol/L) and nPG(3.09 ± 0.73 mmol/L). But after six(T_1_) and twelve hours(T_1_), lactate in prolong group was higher than no-prolong group(T1: PG/nPG, 3.13 ± 0.67/2.62 ± 0.59 mmol/L, *P =* 0.009; T2: PG/nPG, 3.25 ± 0.44/2.02 ± 0.42 mmol/L, *P <* 0.001).

Compared to lactate, PPI showed earlier indication: the value was lower in PG than nPG at T_0_ (1.09 ± 0.33 vs 1.41 ± 0.45, *P =* 0.001) as well as T_1_(1.08 ± 0.37 vs 1.49 ± 0.41, *P <* 0.001). But the difference disappeared after 12 h (T_2_, PG/nPG: 1.17 ± 0.27/1.23 ± 0.33, *P =* 0.392). More information was presented in Table 3.

### Univariate and multivariable analysis

Univariate regression analysis was performed for all potential risk factors of prolonged ICU stay. Only two variables were found significantly related: PPI (T_0_, T_1_), lactate (T_1_, T_2_). More details showed in Table 4. Because T_1_ was the only time point at which both PPI and lactate were significantly associated with prolonged ICU stay, we performed the multivariable logistic regression using PPI_T1_ and lactate_T1_.

**Table 4:** Univariate logistic regression analysis for possible risk factors of prolonged ICU stay

Variable	B	SE	Wald	P	OR		95% CI for OR	
							Lower	Upper
Univariate								
Age /y		−0.028	0.025	1.212	0.271	0.973	0.926	1.022
Gender		0.455	0.355	1.641	0.200	1.576	0.786	3.162
SOFA		0.080	0.171	0.220	0.639	1.084	0.775	1.516
APACHE II		0.153	0.184	0.692	0.406	1.166	0.812	1.672
Surgical length /ml		0.007	0.004	2.671	0.102	1.007	0.999	1.015
Intraoperative fluid infusion		−0.001	0.001	1.113	0.291	0.999	0.998	1.000
Fluid balance /ml	0-6 h	−0.001	0.002	0.265	0.606	1.001	0.997	1.005
	0-12 h	−0.002	0.001	1.809	0.179	0.998	0.995	1.001
HR bpm	T0	−0.006	0.024	0.054	0.816	0.994	0.948	1.043
	T1	−0.020	0.043	0.226	0.634	0.980	0.900	1.066
	T2	−0.009	0.035	0.063	0.802	0.991	0.925	1.062
MAP /mmHg	T0	−0.013	0.033	0.165	0.685	0.987	0.926	1.052
	T1	−0.021	0.049	0.180	0.671	0.979	0.890	1.078
	T2	0.034	0.042	0.651	0.420	1.034	0.953	1.123
PPI	T0	−2.088	0.701	8.866	***0.003***	0.124	0.031	0.490
	T1	−2.586	0.734	12.424	***< 0.001***	0.075	0.018	0.317
	T2	−1.296	0.850	2.327	0.127	0.274	0.052	1.447
Lactate /mmol. L^−1^	T0	−0.009	0.334	0.001	0.978	0.991	0.515	1.907
	T1	1.023	0.407	6.331	***0.012***	2.783	1.254	6.175
	T2	4.645	1.000	21.575	***< 0.001***	104.103	14.662	739.179
T /°C	T0	−0.039	0.718	0.003	0.957	0.962	0.236	3.929
	T1	1.689	1.221	1.912	0.167	5.412	0.494	59.257
	T2	0.365	0.574	0.405	0.524	1.441	0.468	4.441

The multivariable logistic regression analysis demonstrated that lactate_T1_(OR 3.216; 95% CI 1.253–8.254, *P* = 0.015) and PPI_T1_ (OR 0.070; 95% CI 0.016–0.307, *P* < 0.001) were independent predictors of prolonged ICU stay in surgical patients. In Table 5, more details of the logistic regression model were showed.

**Table 5:** Multivariate logistic regression analysis for possible risk factors of prolonged ICU stay

Variable	B	SE	Wald	P	OR	95% CI for OR	
						Lower	Upper
PPI _T1_	−2.666	0.758	12.379	< 0.001	0.070	0.016	0.307
Lac _T1_	1.168	0.481	5.901	0.015	3.216	1.253	8.254

### ROC analyses

**Fig. 2:**
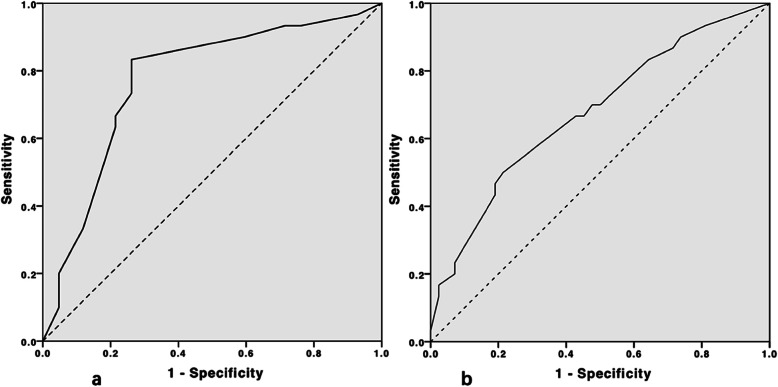
The ROC curves of PPI_T1_ and lactate_T1_ for prolonged ICU stay in surgical patients transferred to ICU. **a**: The ROC curve of PPTT1 in predicting prolonged ICU stay. The dotted line is reference line; solid line is PPTT1; **b**: The ROC curve of lactateT1 in predicting prolonged ICU stay; The dotted line is reference line

ROC curves were drawn to compare the predictive values of PPI_T1_, lactate_T1_ for prolonged ICU stay (Fig. 2). The AUC (area under curve) demonstrated that the predictive values of PPI_T1_ and lactate_T1_ were 0.772 (95% CI: 0.658–0.886) and 0.677 (95% CI, 0.550–0.803) (Table 6). The cutoff values for PPI_T1_ and lactate_T1_ were 1.35(sensitivity: 83.3%; specificity: 73.8%) and 3.10 (sensitivity: 50%; specificity: 78.6%) respectively, based on the maximum Youden index.

**Table 6:** The results of ROC analysis of lactate T_1_ and PPI T_1_ predicting prolonged ICU stay

Variables	ROC area	95% CI	Cutoff value	Sensitivity	Specificity
PPI T_1_	0.772	0.658–0.886	1.35^c^	83.3%	73.8%
Lac T_1_	0.677	0.550–0.803	3.10^d^	50.0%	78.6%

^a^PPI_T1_ < 1.35, predicting prolonged ICU stay with 83.3% sensitivity and 73.8% specificity

^b^Lactate_T1_ > 3.10 mmol/L, predicting prolonged ICU stay with 50.0% sensitivity and 78.6% specificity

## Discussion

The most important finding of this research is association between PPI and length of ICU stay. If PPI can reach 1.35 within the first 6 h in ICU, it gives physicians a hint that this patient could discharge from ICU within 48 h, with 83.3% sensitivity and 73.8% specificity. Besides, we also proved that PPI indicated prolonged ICU stay more superior and earlier than lactate.

### Why PPI can predict prolonged ICU stay and is superior to lactate

Peripheral perfusion index (PPI) is an indicator mirrorring inadequate perfusion in critical patients [7]. It is measured using pulse co-oximetry technology which is characterized by being real-time and non-invasive. PPI is defined as “the ratio of pulsatile blood flow to the non-pulsatile blood flow”, mirroring the strength of blood flow and quality of perfusion at sensor site, reflecting perfusion state of the body part [9, 10]. Compared to blood lactate testing, PPI is real-time and non-invasive [9].

Many researches have proved quality of perfusion is essential for critical patients in the process of recovery [2, 8, 11, 12]. Poor perfusion prolongs patients’ in-hospital stay and increases mortality and morbidity [11]. That is why PPI, a regional perfusion indicator, is related to length of ICU stay. Lower PPI means poorer perfusion and longer ICU stay. But there is another question: why PPI is superior to lactate, an index that has been widely validated and used. Before we figure it out, we need to know alterations of blood flow during hypoperfusion.

At the initial stage of hypoperfusion, peripheral vessels contract in order to return enough blood to heart [13, 14]. In this phase, macro vital signs, like HR and BP, are normal. Because macro circulation is stable, blood lactate does not increase. But PPI, an indicator of regional perfusion, will decrease because vascular contraction reduces reginal blood flow. This makes PPI superior to other macro parameters, for example lactate, in alerting physicians to hypoperfusion.

We can see this superiority of PPI from our results. At T_0_, PPI showed significant differences between PG and nPG, but lactate was not statistically different. After 6 h, lactate became significantly different between PG and nPG at T_1_. There was a delay in lactate comparing to PPI. In order to find out more details about tendency of lactate in two groups, we performed Wilcoxon signed-rank test (abnormal distribution) and found out that lactate showed no obvious decreasing trend in prolonged group while dramatically reduced in non-prolonged group (see supplement3). PPI was higher in nPG than PG both at T_0_ and T_1_. Considering that PPI has been proved effective in reflecting perfusion state, the different tendencies in two groups probably result from PPI. From the.

In order to confirm lactate is not real-time enough to predict poor prognosis, we performed a similar multivariable logistic regression (see supplement 4). In that model, lactateT_0_ is not associated with prolonged ICU stay while PPI_T0_ is relevant with it. We also plotted ROC curves of PPI_T1_ and lactate_T1_ for predicting prolonged ICU stay. The AUC of PPI_T1_ and lacatate_T1_ are 0.772 and 0.677 respectively. These results showed us that PPI predict prolonged ICU stay earlier and better.

### The role of PPI in managing postoperative patients’ treatment

Patients undergoing long time surgeries tend to manifest high level of blood lactate [15, 16]. It is known to us all that hyperlactacidemia is a strong indicator for poor prognosis [12, 17]. If we want to reduce blood lactate, we need to ensure adequate perfusion. According to our conclusions, intensivists should pay more attention to PPI, an invasive and real-time indicator, which has been proved superior to lactate in predicting patients’ prognosis. In a previous study [18], PPI < 1.4 is a sign of hypoperfusion in critical patients. Our conclusion is that PPI < 1.35 after 6 h in ICU predicts LOS ICU longer than 48 h with 83.3% sensitivity and 73.8% specificity, which is similar to Lima AP’s study [18].

In clinic practice, intensivists should be aware of postsurgical patients’ alterations of PPI. If PPI shows obvious trend of declination, doctors should be cautious of hypoperfusion and poor prognosis. They need to find out the reason of its decrease and rule out non-perfusion relevant factors, trying to restore adequate perfusion.

### Factors that influence PPI

One of the reasons limiting the use of PPI is that it is easily influenced by other factors. Low temperature and vascular diseases are the two main confounding factors which could affect value of PPI [18]. Besides, the position can also affect PPI [19]. If we place patients’ arms on unsuitable positions, for example, placing arms under a weight, it will have negative effects on regional perfusion.

Before we apply PPI into clinic practice, we need to keep in mind that lower value does not mean poor perfusion unless we rule out other confounding factors. In this study, we excluded all patients with diseases affecting regional blood flow. In our routine nursing, we give warm-keeping service to all the patients, for example covering blankets. If necessary, we provide warm blower to maintain patients’ body temperature. Usually, we do not place any weights on patients’ arms and all patients are in supine positions unless they require special treatments, for example prone position for acute respiratory distress syndrome. All patients enrolled in our study were supine.

### What is new about PPI in our study compared to previous ones

Many studies proved PPI’s predictive role in hypoperfusion [6, 7, 20] and its association with mechanically ventilated patients’ mortality [8]. What is novel in our study is that we compared PPI with lactate, which has not been showed in previous study and defined another important aspect of PPI: association with length of ICU stay.

Before our study, there is an important research carried out by Van Genderen ME [20] about exploring the superiority of different peripheral perfusion indicators. In that study [20], the siginificance of parameters relecting the state of peripheral perfusion in predicting the occurrence of post-operative complications had been proved. But it did not compare these indicators with the most widely used parameters relefcting perfusion: blood lactate. If we want to popularize a new parameter, we must compare its property with the extensively used one. That is why we perform our study, and luckily, we proved PPI’s strenghth over lactate.

In addition to that study, there is another research, written by Su L etc. [8], about the role of PPI in predicting mortality of patients with mechanical ventilation. Unlike Su’s research [8], mainly focusing on patients with respiratory failure requiring mechanical ventilation, our study is about applying PPI in postoperative management.

### Limitations

This study has several limitations. First, its sample size is small. More studies on PPI with bigger sample size should be carried out in the future. Second, patients who died in ICU or discharged against medical advice were excluded. This would cause the patients included in our study less severe. As we can see from the results, most patients’ PPI could reach to around 1.2 within 12 h. Besides, the highest average blood lactate in both groups was less than 4.0 mmol/L. These phenomena indicate patients enrolled in the study were not so serious. It is better to confine our conclusions to surgical patients who are not in life-threatening conditions. As for patients on the edge of death, predicting their LOS ICU is much more complicated. Third, our studying period is only 12 h. The reason why we focus on a short time is that we want to foresee patients’ outcomes in early clinic phase. It is worth to note that many complications could appear in the late stage, these complications may postpone the discharge. A previous study had confirmed PPI alteration was associated with development of severe complications [20]. So, we have confidence that decreased PPI is associated with prolonged ICU stay even in the long term.

## Conclusions

For surgical patients transferred to ICU, PPI and lactate at T1(6 h after ICU admission) are associated with length of ICU stay. Compared to lactate, PPI reflects prolonged ICU stay earlier and better than lactate. The value of PPI at T1 lower than 1.35 has 83.3% sensitivity and 73.8% specificity in predicting LOS ICU longer than 48 h.

## Supplementary information

**Additional file 1.**

**Additional file 2.**

**Additional file 3.**

## Data Availability

The datasets generated and/or analyzed during the current study are not publicly available due the security of our patients, but are available from the corresponding author on reasonable request.

## Abbreviations

PPI: Peripheral perfusion index
SOFA: Sequential Organ Failure Assessment
APACHE II: Acute Physiology and Chronic Health Evaluation
HR: Heart rate
MAP: Mean artery pressure
ICU LOS: Length of ICU stay
T: Temperature
CI: Confidence interval
CC: Correlation coefficient
ROC: Receiver operating characteristic
AUC: Area under curve
CAD: Coronary artery disease
COPD: Chronic obstructive pulmonary disease
CKD: Chronic kidney disease
HTN: Hypertension
PMI: Pacemaker implantation
GO: Gynecological oncology
GA: Great artery
