# Supplementary material for: Peripheral perfusion index predicting prolonged ICU stay earlier and better than lactate in surgical patients: an observational study
Source: BMC Anesthesiol. 2020 Jun 18;20:153. doi: 10.1186/s12871-020-01072-0 (PMC7301460; doi:10.1186/s12871-020-01072-0)
Supplement: Supplementary file 1 — Additional file 1. [file 12871_2020_1072_MOESM1_ESM.docx]

This study is a retrospective case-control study. The case group is “prolonged ICU stay” and control group is “non-prolonged ICU stay”. The main indicator of interest is peripheral perfusion index. With reference to a previous research on PPI [1], we set the value of PPI in case group as 2.7±0.8(D0, group “severe complications”), in control group as 3.5±0.4(D0, group “non-severe complications”).

Other parameters setting:

a) Sampling ratio is 1:1;

b) The power (1-β) is 0.9;

c) Type I error rate(α) is 0.05.

PASS 2020 (© 2013-2020 HyLown Consulting LLC • Atlanta, GA) was used to calculate the sample size. The result was ***fourteen patients*** in each group minimally.

**Reference**

1. van Genderen, Michel E et al. “Clinical assessment of peripheral perfusion to predict postoperative complications after major abdominal surgery early: a prospective observational study in adults.” Critical care, 2014; 18(3): R114
