# Supplementary material for: Peripheral perfusion index predicting prolonged ICU stay earlier and better than lactate in surgical patients: an observational study
Source: BMC Anesthesiol. 2020 Jun 18;20:153. doi: 10.1186/s12871-020-01072-0 (PMC7301460; doi:10.1186/s12871-020-01072-0)
Supplement: Supplementary file 2 — Additional file 2. [file 12871_2020_1072_MOESM2_ESM.docx]

| Potential active hemorrhage | Internal environment disturbance | Disturbance of consciousness | Suspicious infection | Prolonged mechanical ventilation | higher lactate | sudden oliguria | Myocardial damage | Unclear |
| --- | --- | --- | --- | --- | --- | --- | --- | --- |
| 7 | 10 | 6 | 9 | 7 | 12 | 3 | 3 | 8 |

Reasons for prolonged ICU stay

Explanations and definitions:

***Internal environment disturbance:***

Hyperkalemia：4

Acidosis (excluded hyperkalemia associated acidosis):3

Alkalosis:2

Hypokalemia:1(one patient, with post gastrectomy, lower than 3.0mmol/L)

***Disturbance of consciousness:***

Drowsiness:3

Delirium:2

Lethargy:1(this patient was found out to be focal cerebral infarction)

***Suspicious infection***

We defined it as applying broad spectrum antibiotics and tested etiology specimens. Prophylactic application of antimicrobial was not attributed to suspicious infection. We got all the information from e-medical records.

***Prolonged mechanical ventilation:***

We defined it as the length of MV longer than 48h.

***Hypoperfusion*** (we divided this part into two new groups: higher lactate and sudden oliguria, because not all sudden oliguria results from inadequate renal perfusion).

**Higher lactate:** If patient’s lactate is higher than 2.0mmol/L, we will defer his/her discharge from ICU and spend more time on finding out the reason behind hyperlactacidemia. But if his/her baseline of lactate is high, for example patient diagnosed with lymphoma, we will transfer his/her to general ward normally.

**Sudden oliguria:** if urine volume less than 0.5ml/kg.h-1 suddenly occurs, lasting more than 3h, we will postpone patients’ discharge from ICU to detect the cause of oliguria.

***Myocardial damage:***

Newly elevated troponin with or without dynamic ECG change.
