# Supplementary material for: Peripheral perfusion index predicting prolonged ICU stay earlier and better than lactate in surgical patients: an observational study
Source: BMC Anesthesiol. 2020 Jun 18;20:153. doi: 10.1186/s12871-020-01072-0 (PMC7301460; doi:10.1186/s12871-020-01072-0)
Supplement: Supplementary file 3 — Additional file 3. [file 12871_2020_1072_MOESM3_ESM.docx]

| Variable | B | SE | Wald | P | OR | 95% CI for OR | |
| --- | --- | --- | --- | --- | --- | --- | --- |
|  |  |  |  |  |  | Lower | Upper |
| Multivariate | | | | | | | |
| Gender | 0.298 | 0.554 | 0.289 | 0.591 | 1.347 | 0.455 | 3.988 |
| Surgical length | 0.007 | 0.005 | 2.407 | 0.121 | 1.007 | 0.998 | 1.017 |
| Fluid input in 1st 24h * | 0.639 | 0.519 | 1.515 | 0.218 | 1.895 | 0.685 | 5.246 |
| PPI T0 | -1.955 | 0.717 | 7.444 | ***0.006*** | 0.142 | 0.035 | 0.577 |
| Lac T0 | 0.061 | 0.375 | 0.026 | 0.871 | 1.063 | 0.509 | 2.219 |
